# Effects of electrical muscle stimulation on core muscle activation and physical performance in non-athletic adults: A randomized controlled trial

**DOI:** 10.1097/MD.0000000000032765

**Published:** 2023-01-27

**Authors:** Hyun-Joon Yoo, Sangsoo Park, Sejun Oh, Munjeong Kang, Yongha Seo, Byung Gon Kim, Sang-Heon Lee

**Affiliations:** a Department of Physical Medicine and Rehabilitation, Korea University Anam Hospital, Korea University College of Medicine, Seoul, Republic of Korea; b School of Global Sport Studies, Korea University Sejong Campus, Sejong City, Republic of Korea; c Human Behavior & Genetic Institute, Associate Research Center, Korea University, Seoul, Republic of Korea; d Korea Health Exercise Manager Association, Seoul, Republic of Korea; e QOLFIT Training Center, Seoul, Republic of Korea.

**Keywords:** abdominal muscle, electrical stimulation, exercise, muscle contraction, strength training

## Abstract

Electrical muscle stimulation (EMS) activates muscles through electrical currents, resulting in involuntary muscle contractions. This study aimed to evaluate the immediate clinical effects of superimposing EMS on strength training compared with conventional exercise in healthy non-athletic adults.

**Methods:**

This study was a randomised, controlled, parallel-group trial conducted at a single centre. Forty-one healthy young volunteers were recruited and randomised into two groups: strengthening with superimposed EMS (S+E) and strengthening (S) groups. All participants underwent the 30 minutes of strength training program, three times a week for 8 weeks, consisting of core muscle exercises. Additionally, the S+E group received EMS during training, which stimulated the bilateral abdominal, gluteus, and hip adductor muscles. As the primary outcome measure, we evaluated the changes in muscle thickness, including the abdominal, gluteal, and hip adductor muscles, using ultrasound. Muscle thickness was measured in both resting and contracted states. For secondary outcomes, physical performance (Functional Movement System score, McGill’s core stability test, and hip muscle power) and body composition analysis were evaluated. All assessments were performed at the beginning and end of the intervention.

**Results:**

39 participants (S+E group = 20, S group = 19) completed the study. The clinical characteristics and baseline functional status of each group did not differ significantly between the groups. After completion of the training, the S+E group showed more efficient contraction in most of the evaluated muscles. The resting muscle thickness did not differ significantly between the groups; however, the contracted muscle thickness in the S+E group was higher than that in the S group (p < 0.05). Physical performance and body composition were not significantly different between the two groups. No intervention-related complications were reported during the study.

**Conclusion:**

EMS seems to be a safe and reasonable modality for improving physical fitness in healthy individuals.

## 1. Introduction

Electrical muscle stimulation (EMS) has long been used as a complementary training method, applied either locally^[1,2]^ or to the whole body.^[3,4]^ It activates muscles artificially through various electrical current forms, which are delivered through electrodes on the target muscles. As a result, electrical current induces involuntary muscle contraction which produces similar exercise benefits without much discomfort.^[5]^ More specifically, it was designed to facilitate passive activation of a large number of motor units and induce synchronous recruitment of muscle fibers, with the aim of strengthening or maintaining muscle mass.^[6–8]^ Owing to recent advances in EMS technology, relatively low-cost and portable EMS devices have been developed and applied in several settings (e.g., hospitals, clinics, homes, and leisure sports).

Previous studies have demonstrated the effectiveness of EMS on muscle function and physical performance in various populations including healthy young individuals, the elderly, and patients with debilitating muscle-wasting conditions such as sarcopenia.^[1,2,9–13]^ In particular, EMS is a feasible and effective rehabilitation approach for patients with impaired physical performance or for older people with low physical activity. Although conventional resistance exercises are the most recommended intervention for the management of muscle health, factors such as physical limitations, time constraints, and low motivation disturb the engagement of such voluntary exercises.^[9]^ In such situations, EMS presents an opportunity to increase adherence to an exercise program^[10]^ and improve body composition and physical strength for patients.^[4]^ Investigators have reported that EMS improves quadriceps strength and lower extremity function in frail older patients with acute heart failure,^[11]^ preserves muscle mass and function in patients with sarcopenia,^[12]^ improves balance and reduces fall risk among the elderly,^[13]^ reduces deltoid atrophy after arthroscopic rotator cuff repair,^[14]^ and improves quadriceps strength and decreases pain in patients with knee osteoarthritis.^[15]^ However, there are few studies on the effectiveness of EMS, especially in patients in intensive care settings, which requires further research.^[8,16]^

EMS training has also been widely used in healthy individuals and implemented in competitive athletes.^[17]^ Some studies have examined the training effects of EMS in elite sports players such as rugby, ice hockey, and basketball, and reported its positive effects on muscle strength and skilled performance.^[18–20]^ Furthermore, EMS research has been conducted with untrained people. In recent studies, local EMS application can effectively activate superficial abdominal muscles^[21]^ and enhance the cross-sectional area of the lateral abdominal wall and rectus abdominis, as well as lumbopelvic control in healthy subjects.^[22]^ In addition, Nishikawa et al proved that EMS can induce different distributions of quadriceps muscle activation at 50% and 70% of maximal voluntary contraction measured by surface electromyography.^[23]^ A systematic review and meta-analysis revealed that it seems to have positive effects on muscle mass and strength parameters, but not on body fat mass.^[4]^ However, the reported effects of EMS are partially compromised by factors such as the pre-trained status of the subjects, lack of standardization of methods, or intervention protocols. For instance, a recent study compared the effects of EMS, conventional strength training, and superimposing EMS on conventional exercise training (STEMS) on elbow flexor muscle thickness after 8 weeks. In contrast to previous studies, no significant differences were observed among the intervention protocols.^[1]^

In spite of many previous studies, it is reasonable to think that EMS should not be regarded as a replacement for conventional exercise training per se, since the exercise itself enhances not only muscle function, but also exerts positive effects on endothelial function, cardiopulmonary fitness, and cognitive function.^[24]^ Therefore, researchers have focused on the additional benefits of STEMS in healthy individuals. However, only a few studies comparing STEMS and EMS have been conducted, and the effect of STEMS on healthy individuals and additional effects of EMS on conventional exercise training programs remain controversial.^[1,24]^ This study aimed to evaluate the immediate clinical effects of STEMS compared to conventional exercise in healthy non-athletic adults through the analysis of muscle thickness, various physical performance abilities, and body composition during 8 weeks of physical training. To maximize the validity of the study, we conducted a randomized controlled trial with university students of similar age groups using a well-controlled exercise intervention. In addition, we analyzed the effects of EMS on 3 aspects: physical performance, muscle thickness, and body composition. We hypothesized that STEMS would have additional benefits compared to conventional ST, since EMS could induce additional muscle fiber recruitment and generate greater adaptations after training.

## 2. Methods

This study was a single-blinded (outcome assessor), prospective, randomized controlled trial using a parallel group design conducted in a single center from January 2022 to February 2022. Forty-one healthy young volunteers were recruited and randomized into 2 groups: a group (n = 21) that performed 8 weeks of strengthening exercise with EMS (S + E group) and a group (n = 20) that performed only 8 weeks of strengthening exercise (S group). A computer-generated list of random allocations was used, and the randomization results were blinded to the assessment staff. Changes in muscle thickness on ultrasound, including the abdominal, gluteal, and hip adductor muscles were selected as the primary outcome measure. For secondary outcomes, changes in physical performance and body composition were evaluated. All assessments were performed twice: before the intervention and after 8 weeks of intervention.

### 2.1. Participants

**Figure 1.**
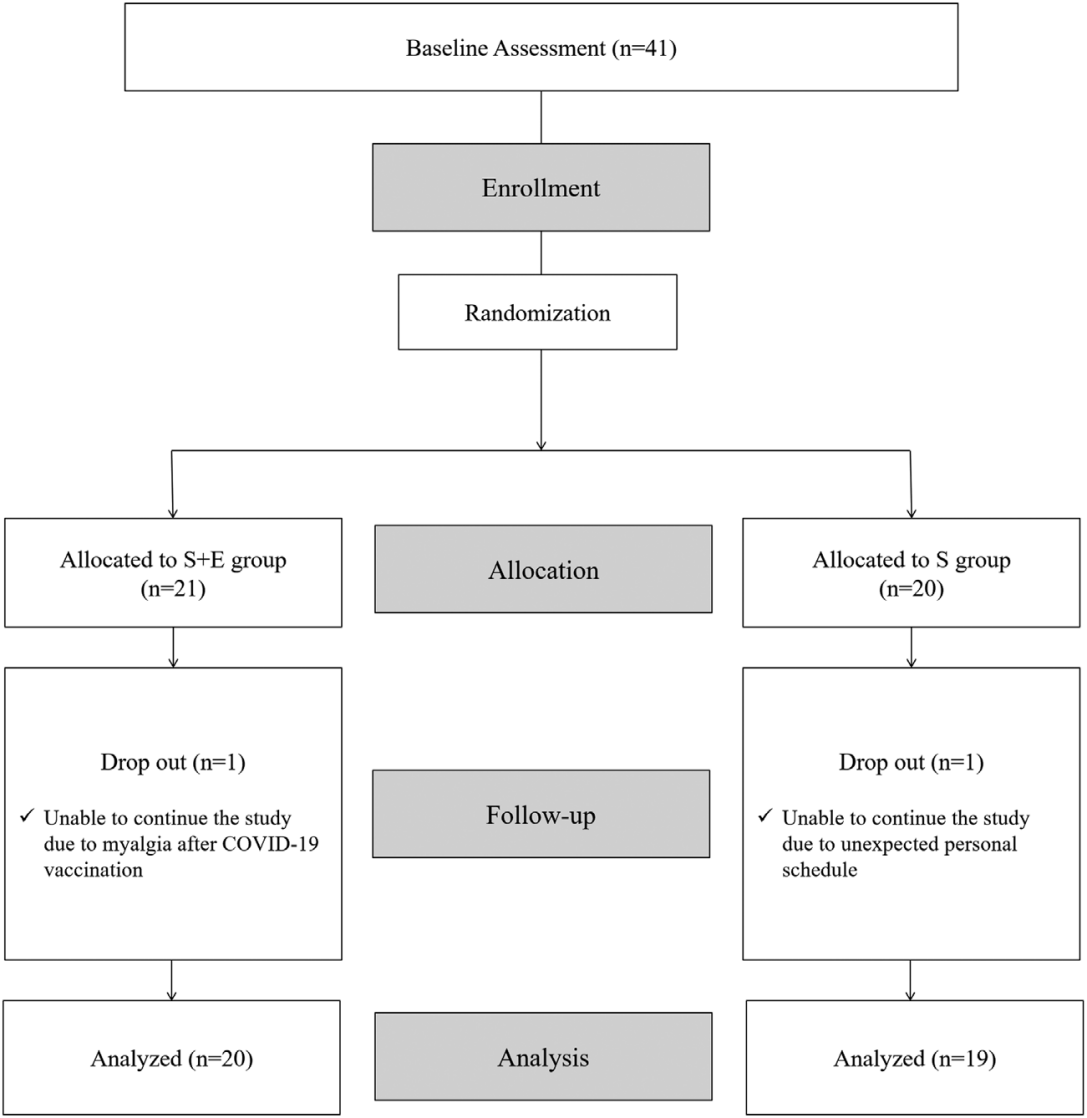
Flowchart of the study.

The sample size was calculated using the Gpower 3.1 program. For an effect size of 0.25, error probability of 0.05, and power of 0.90, 38 participants were defined. Considering the 10% dropout rate, we recruited 41 healthy subjects (male = 17, female = 24). The inclusion criteria were as follows: age between 20 and 25 years, not engaged in elite sports, lack of any history of spinal surgery or persistent low back pain, lack of any medical conditions that affect muscle metabolism, such as myopathy, and lack of any medical history that might be affected by EMS, such as epilepsy or cardiac pacemaker insertion. All participants provided written informed consent prior to participation. The study was approved by the Institutional Review Board of Korea University Anam Hospital (2021AN0557). A CONSORT diagram is shown in Figure 1.

### 2.2. Strength training protocol

During the intervention period, all participants performed 30 minutes of strength training, 3 times per week (Monday, Wednesday, and Friday) for 8 weeks at the Jangan University fitness gym. Each exercise program included an additional 5 minutes of warm-up and cool-down periods, with a rest time of 30 seconds between each set. For the first 2 weeks, the training focused on the core muscle stabilizing exercise, and for the remaining 6 weeks, the training was mainly intended to strengthen the core muscles. More specifically, the first 2 weeks focused on controlling core muscle contraction and relaxation with open kinetic exercises. Then, we gradually increased the exercise intensity with closed kinetic exercise, such as a plank for the period from the 3rd to 5th weeks. Finally, the 6th to 8th weeks of the exercise program were used for more functional exercises, such as mini squats, to incorporate and maximize the related core muscle function. Exercise intensity was adjusted based on the Borg category ratio (CR)-10 scale.^[25]^ The rate of perceived exertion was gradually increased from “easy” to “very hard” (Borg CR-10 scale “2” to “9”) during the 8 weeks of intervention. The detailed physical training program is described in Table, Supplemental digital content, http://links.lww.com/MD/I379. The participants were allowed a sufficient warm-up and cool-down period before and after exercise. All the training sessions were guided and supervised by a professional athletic trainer (YHS). Additional regular exercises were not allowed during the intervention period to minimize confounding factors.

### 2.3. Electrical muscle stimulation

**Figure 2.**
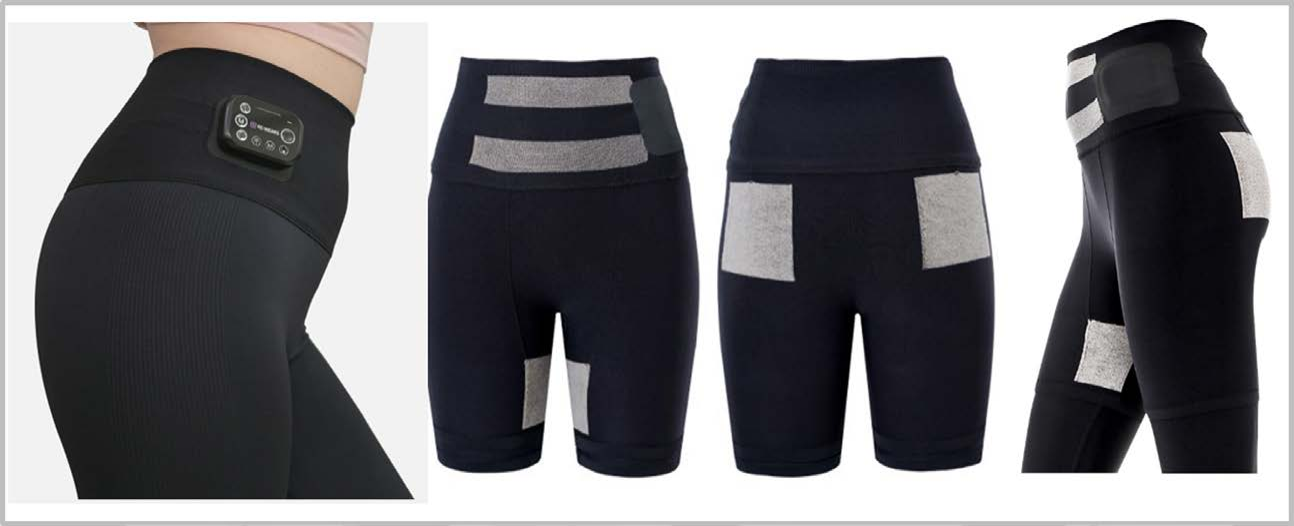
The wearable EMS training pants and positioning of the electrodes. EMS = electrical muscle stimulation.

In this study, we used a wearable EMS training pant (Rewears EMS training pants, SP COMPANY, Seoul, Korea) during strength training in the S + E group. It was designed to simultaneously stimulate the bilateral abdominal, gluteus medius, and hip adductor muscles (Fig. 2). In particular, the EMS pads were fabricated using a high-conductivity silver thread so that they could efficiently deliver electrical current to the target muscles. The device was allowed to modulate the stimulation intensity by alternating the stimulation frequency from 8 to 70 Hz. Wearable EMS training pants were available in various sizes. During the 1^st^ week of the training period, we applied 30 Hz EMS stimulation for adaptation, and 70 Hz EMS stimulation was applied during the rest of the training period. Although there are some scientific arguments about the optimal electrical stimulation frequency, it is generally accepted that high-frequency stimulation generates greater neuromuscular adaptations at the level of fast-twitch fibers and enhances the strength of the target muscles.^[26]^ Therefore, if the intensity of electrical stimulation is not sufficiently high, the effects of STEMS are less efficient in restoring muscle strength and cross-sectional area.^[27]^ A recent guideline for EMS parameters concluded that an EMS frequency between 50 and 75 Hz is the most effective and should not exceed 75 Hz.^[15]^ Therefore, we used 70 Hz EMS stimulation for most of the study period.

### 2.4. Outcome measurements

#### 2.4.1. Muscle thickness.

All sonographic images were collected by a single-blinded experienced sonographic operator (HJY). We evaluated the thickness of the rectus abdominis (RA), external oblique (EO), internal oblique (IO), transverse abdominis (TrA), gluteus medius (GMed), and adductor longus (AL) on the dominant side. All muscles were investigated in both resting and contracted states. Ultrasound (US) images were acquired in B-mode with a portable US machine (MX7, Mindray, NJ) using a linear (3–12.8 MHz, L12-3RCs Transducer, Mindray, NJ) probe for abdominal muscles and a convex probe (1.4–5.1 MHz, C5-1s Transducer, Mindray, NJ) for GMed and AL.

Transducer placement and participant position were determined based on previous studies.^[28–30]^ More specifically, the resting thickness of the 4 abdominal muscles (RA, EO, IO, and TrA) was obtained in the supine hook-lying position with arms at the side and head in the midline. Then, contracted thicknesses were measured during curl-ups for the RA and the abdominal drawing-in maneuver for the EO, IO, and TrA. For the RA, 1 side of the transducer was placed immediately above the umbilicus and the greatest perpendicular thickness between the superficial and deep fascial layers was measured; for the EO, IO, and TrA, the transducer was just superior to the iliac crest along the mid-axillary line. Then, the thickness of each muscle was measured perpendicularly between adjacent fascial borders in the middle of the images. All abdominal muscles were evaluated at the end of normal expiration. The resting thickness of the GMed was obtained from a side-lying position, and the contracted thickness was measured during the hip abduction task. The probe was located on the lateral aspect of the hip on the lower half of a coronal line located between the top of the greater trochanter and 25% of the distance between the anterior superior iliac spine and the posterior superior iliac spine. Finally, the resting thickness of the AL was obtained in the side-lying position, and the contracted thickness was measured during the hip adduction task. The probe was located on the medial anterior aspect of the thigh, between the lateral condyle of the femur and greater trochanter. All subjects were evaluated under the same conditions, and all US settings were kept unchanged throughout the experimental period.

#### 2.4.2. Physical performance.

Physical performance was evaluated using the functional movement screen (FMS) score, McGill’s core stability test, and hip muscle power. The FMS is designed to assess functional movement abilities and asymmetries which reflect general musculoskeletal conditions. It consists of 7 fundamental movement tests, each scored on a scale of 0 to 3 and a composite score ranging from 0 to 21 points. The 7 movement tests were deep squat, in-line lunge, hurdle step, shoulder mobility, active-straight leg raise, trunk stability pushup, and quadruped rotary stability.^[31,32]^ McGill’s core stability test is used to assess core stability and consists of the isometric extensor, isometric flexor, and side-bridge exercise tests.^[33]^ The subjects were encouraged to maintain isometric postures for each test as long as possible, and the length of time was recorded in seconds. Hip muscle power was measured using a dynamometer (MicroFET2; Hogan Health Industries, UT). All force measurements were acquired using isometric tests: hip flexion and extension, hip internal and external rotations, and hip abduction and adduction. The measurements were performed twice, and the values were averaged for each test. All physical performances were evaluated by a blinded athletic trainer (YHS).

#### 2.4.3. Body composition analysis.

Body composition was analyzed using bioelectrical impedance analysis (BIA) (InBody 570, Inbody Co., Ltd., Seoul, Korea). The device splits body mass into 3 components: fat, muscle, and bone mineral. We evaluated changes in whole-body fat and skeletal muscle mass. Additionally, we evaluated the InBody score before and after the intervention, which reflects the overall evaluation of body composition. The higher the muscle mass, the higher is the score.

### 2.5. Statistical analyses

Parametric statistics were used because all the data in the study showed a normal distribution in the Shapiro–Wilk test (*P* < .05). Baseline characteristics were compared using the independent t-test for continuous variables and chi-square test for categorical variables. In addition, the paired *t* test was used for within-group analysis, and the independent *t* test was used for between-group analysis after the completion of the training. The effect size values were further calculated and the strength of the mean difference for each *t* test and chi-square test was quantified by Cohen’s d (small effect: 0.2, medium effect: 0.5, large effect: 0.8) and Cramér’s V effect size (small effect: 0.1, medium effect: 0.3, large effect: 0.5), respectively. For all tests, statistical significance was set at *P* < .05. All statistical analyses were conducted using SPSS software (SPSS version 29.0; SPSS Inc., Armonk, NY).

## 3. Results

### 3.1. Baseline

Among the 41 participants, 39 (20 in the S + E group and 19 in the S group) completed all evaluations and the intervention. One subject in the S + E group dropped out because of myalgia after COVID-19 vaccination, and 1 subject in the S group dropped out because of an unexpected personal schedule. The baseline demographic and clinical characteristics of the study participants are summarized in Table 1. No significant differences were noted between the 2 groups including baseline characteristics, muscle thickness, physical performance, and body composition. Only the thickness of the contracted gluteus medius was significantly different between the groups (*P* = .02).

**Table 1:** Baseline characteristics of the study participants (N = 39).

	S + E group (N = 20)	S group (N = 19)	*P* value (effect size)
Demographic information			
Sex (male/female)	7/13	8/11	0.65 (0.07)
Age (yr)	23.40 ± 2.28	23.10 ± 1.88	0.66 (−0.14)
Body mass index	23.06 ± 4.84	25.37 ± 6.52	0.22 (0.40)
Resting muscle thickness			
RA (cm)	1.13 ± 0.23	1.29 ± 0.32	0.08 (0.59)
EO (cm)	0.85 ± 0.29	0.93 ± 0.38	0.43 (0.26)
IO (cm)	0.88 ± 0.32	1.05 ± 0.32	0.11 (0.53)
TrA (cm)	0.51 ± 0.16	0.53 ± 0.22	0.67 (0.14)
GMed (cm)	2.28 ± 0.48	2.64 ± 0.64	0.06 (0.65)
AL (cm)	4.63 ± 1.01	4.89 ± 1.51	0.53 (0.20)
Contracted muscle thickness			
RA (cm)	1.43 ± 0.33	1.67 ± 0.55	0.12 (0.52)
EO (cm)	1.51 ± 0.56	1.50 ± 0.69	0.97 (−0.01)
IO (cm)	1.35 ± 0.54	1.47 ± 0.52	0.49 (0.23)
TrA (cm)	0.82 ± 0.36	0.91 ± 0.25	0.37 (0.29)
GMed (cm)	2.77 ± 0.49	3.24 ± 0.68	0.02* (0.81)
AL (cm)	5.49 ± 1.43	5.69 ± 1.64	0.68 (0.13)
Physical performance			
Hip extensor (kgf)	25.88 ± 11.12	30.52 ± 14.58	0.28 (0.36)
Hip flexor (kgf)	26.45 ± 11.21	30.64 ± 13.28	0.30 (0.34)
Hip internal rotator (kgf)	14.79 ± 5.28	15.67 ± 7.19	0.67 (0.14)
Hip external rotator (kgf)	14.12 ± 5.10	16.12 ± 7.68	0.35 (0.31)
Hip adductor (kgf)	23.65 ± 8.55	26.78 ± 13.45	0.39 (0.28)
Hip abductor (kgf)	30.68 ± 12.03	35.54 ± 15.34	0.39 (0.28)
FMS score	12.80 ± 2.89	12.10 ± 2.49	0.43 (−0.26)
Flexor endurance (s)	63.13 ± 41.49	53.57 ± 32.33	0.45 (−0.25)
Extensor endurance (s)	119.40 ± 54.96	99.31 ± 45.18	0.24 (−0.40)
Side bridge (s)	42.44 ± 24.71	45.60 ± 27.78	0.84 (0.12)
Body composition			
Skeletal muscle mass (kg)	24.75 ± 6.34	27.76 ± 10.20	0.15 (0.48)
Body fat mass (kg)	18.89 ± 7.22	22.59 ± 10.90	0.22 (0.40)
Inbody score	70.45 ± 4.73	71.74 ± 7.69	0.53 (0.20)

Data are expressed as mean ± standard deviation or n unless otherwise indicated.

AL = adductor longus, EO = external oblique, GMed = gluteus medius, IO = internal oblique, RA = rectus abdominis, TrA = transverse abdominis.

* *P* < .05.

### 3.2. Changes in muscle thickness (US)

As shown in Table 2, it was observed that all of the resting muscle thickness did not change significantly after 8 weeks of intervention in S group. On the other hand, the resting muscle thickness of RA, GMed and AL in the S + E group increased significantly. However, in the between-group analysis, there was no significant group difference in changes in resting muscle thickness except GMed, indicating that EMS had no additional effect on resting muscle thickness changes.

**Table 2:** Changes in muscle thickness in resting and contracted state.

	Group	Changes	*P* value for within group analysis (effect size)	*P* value for between group analysis (effect size)
Resting muscle thickness (cm)				
∆ RA	S + E group	−0.14 ± 0.23	.02* (−0.60)	.12 (−0.51)
	S group	−0.01 ± 0.26	.86 (−0.04)	
∆ EO	S + E group	−0.03 ± 0.23	.52 (−0.15)	.35 (0.30)
	S group	−0.15 ± 0.47	.20 (−0.31)	
∆ IO	S + E group	−0.13 ± 0.45	.20 (−0.30)	.09 (−0.57)
	S group	0.07 ± 0.24	.21 (0.30)	
∆ TrA	S + E group	0.01 ± 0.18	.82 (0.05)	.63 (−0.15)
	S group	0.04 ± 0.25	.47 (0.17)	
∆ GMed	S + E group	−0.56 ± 0.67	<.01* (−0.83)	.01* (−0.88)
	S group	−0.01 ± 0.55	.93 (−0.02)	
∆ AL	S + E group	−0.62 ± 0.90	<.01* (−0.68)	.13 (−0.49)
	S group	−0.19 ± 0.81	.31 (−0.24)	
Contracted muscle thickness (cm)				
∆ RA	S + E group	−0.46 ± 0.30	<.01* (−1.52)	<.01* (−1.44)
	S group	0.01 ± 0.35	.93 (0.02)	
∆ EO	S + E group	−0.57 ± 0.25	<.01* (−2.32)	<.01* (−1.02)
	S group	−0.19 ± 0.48	.11 (−0.39)	
∆ IO	S + E group	−0.37 ± 0.43	<.01* (−0.86)	.03* (−0.74)
	S group	−0.06 ± 0.40	.51 (−0.16)	
∆ TrA	S + E group	−0.20 ± 0.31	.01* (−0.65)	.25 (−0.38)
	S group	−0.09 ± 0.29	.21 (−0.30)	
∆ GMed	S + E group	−0.88 ± 0.54	<.01* (−0.83)	<.01* (−1.16)
	S group	−0.23 ± 0.59	.01* (−0.39)	
∆ AL	S + E group	−1.24 ± 1.08	<.01* (−1.14)	.01* (−0.85)
	S group	−0.39 ± 0.90	.08 (−0.43)	

Data are expressed as mean ± standard deviation or n unless otherwise indicated.

Negative values represent increases after training for the within-group comparisons and in the S + E group for the between-group comparisons.

AL = adductor longus, EO = external oblique, GMed = gluteus medius, IO = internal oblique, RA = rectus abdominis, TrA = transverse abdominis.

* *P* < .05.

Regarding the contracted muscle thickness, all the muscle thickness significantly increased after the intervention in the S + E group, while only the GMed in the S group increased significantly. In the between-group analysis, the degree of increased muscle thickness was significantly higher in the S + E group than S group except TrA (*P* value = .25).

### 3.3. Physical performance

In the within-group analysis, most of the hip muscle power improved significantly after 8 weeks of intervention in both groups. In the FMS score analysis, both groups showed significant improvements in the within-group analysis. In addition, in McGill’s core stability test, the subjects in both groups were able to perform most of the tasks for a longer time, which was statistically significant. Only the extensor endurance test in the S + E group did not improve significantly. However, in the between-group analysis, there were no differences in the degree of improvement in any of the physical performance measures except hip external rotator power (Table 3).

**Table 3:** Changes in physical performance.

	Group	Changes	*P* value for within group analysis (effect size)	*P* value for between group analysis (effect size)
∆ Hip extensor (kgf)	S + E group	−7.34 ± 7.27	<.01* (−1.01)	.52 (−0.21)
	S group	−5.72 ± 8.02	<.01* (−0.71)	
∆ Hip flexor (kgf)	S + E group	−2.71 ± 6.02	.06 (−0.45)	.84 (−0.07)
	S group	−2.16 ± 10.20	.38 (−0.21)	
∆ Hip internal rotator (kgf)	S + E group	−5.00 ± 2.98	<.01* (−1.68)	.04* (−0.69)
	S group	−2.83 ± 3.31	<.01* (−0.75)	
∆ Hip external rotator (kgf)	S + E group	−2.27 ± 2.16	<.01* (−1.05)	.52 (−0.27)
	S group	−1.51 ± 3.32	.07 (−0.46)	
∆ Hip adductor (kgf)	S + E group	−4.18 ± 6.60	<.01* (−0.63)	.80 (0.08)
	S group	−4.77 ± 7.79	.02* (−0.61)	
∆ Hip abductor (kgf)	S + E group	−5.64 ± 6.21	<.01* (−0.77)	.08 (−0.59)
	S group	−2.07 ± 5.98	.16 (−0.35)	
∆ FMS score	S + E group	−3.70 ± 2.02	<.01* (−1.82)	.21 (0.41)
	S group	−4.52 ± 2.01	<.01* (−2.25)	
∆ Flexor endurance (s)	S + E group	−60.45 ± 79.56	<.01* (−0.76)	.11 (−0.55)
	S group	−24.63 ± 43.37	.03* (−0.57)	
∆ Extensor endurance (s)	S + E group	−16.00 ± 63.14	.27 (−0.25)	.64 (0.16)
	S group	−24.92 ± 48.03	.048* (−0.52)	
∆ Side bridge (s)	S + E group	−30.23 ± 31.26	<.01* (−0.97)	.16 (−0.48)
	S group	−17.61 ± 18.87	<.01* (−0.93)	

Data are expressed as mean ± standard deviation or n unless otherwise indicated.

Negative values represent increases after training for the within-group comparisons and in the S + E group for the between-group comparisons.

FMS = functional movement screen.

* *P* < .05.

### 3.4. Body composition analysis

Finally, we analyzed the changes in body composition in terms of skeletal muscle mass, body fat mass, and body score (Table 4). Overall, skeletal muscle mass and Inbody scores increased after the intervention in both the groups. Body fat mass decreased in the S + E group. However, most of the changes were not statistically significant in both the within- and between-group analyses in either group.

**Table 4:** Changes in body composition analysis.

	Group	Changes	*P* value for within group analysis (effect size)	*P* value for between group analysis (effect size)
∆ Skeletal muscle mass (kg)	S + E group	−0.14 ± 0.86	.49 (−0.16)	.43 (0.26)
	S group	−0.33 ± 0.66	.04* (−0.50)	
∆ Body fat mass (kg)	S + E group	0.39 ± 1.61	.29 (0.24)	.49 (0.22)
	S group	−0.04 ± 2.20	.94 (−0.02)	
∆ Inbody score	S + E group	−0.40 ± 2.26	.44 (−0.18)	.88 (−0.05)
	S group	−0.26 ± 3.16	.72 (−0.08)	

Data are expressed as mean ± standard deviation or n unless otherwise indicated.

Negative values represent increases after training for the within-group comparisons and in the S + E group for the between-group comparisons.

* *P* < .05.

## 4. Discussion

EMS has been regarded as a bridge to conventional exercises. It has been postulated that EMS superimposed on voluntary contractions would have a significant impact on muscle strength and physical performance. More specifically, STEMS has been known to enhance motor unit recruitment of stimulated muscles, thereby increasing neuromuscular adaptations and motor control.^[26]^ However, the effects of STEMS are not clearly understood because of the lack of well-conducted randomized studies. In particular, few studies have examined the effects of superimposed EMS on core muscles. In this study, we evaluated the effects of STEMS compared to ST in healthy non-athletic adults. In particular, we comprehensively analyzed the effects of EMS on various aspects such as changes in muscle thickness, physical performance, and body composition. The main finding of this randomized controlled study was that 8 weeks of STEMS improved the degree of contracted core muscle thickness more effectively than ST. In addition, both STEMS and ST improved most of the physical performance, even though there was no significant difference between the 2 groups. Besides, the EMS device and exercise protocol in the study was proven to be safe since none of the participants reported discomfort nor did any severe adverse events occur during the study period.

EMS and conventional exercise induce different modes of muscle activation, resulting in different physiological effects and adaptations in the neuromuscular system. During voluntary contraction, motor units are recruited in sequential order from small to large, which is known as the size principle. Physiologically, synaptic currents activate motor unit recruitment, and smaller motor units tend to be activated more easily.^[34]^ In contrast, the EMS activates large motor units before small motor units. The external electrical current of EMS stimulates nerve axon fibers and preferentially large motor units, which have low impedance. In summary, EMS tends to reverse motor unit recruitment compared to conventional exercise. In addition, EMS is more likely to activate the muscles located directly beneath the stimulation electrodes.^[35]^ It has also been postulated that respiratory, cardiac, and metabolic responses during EMS differ from those induced during voluntary muscle contraction.^[36]^ Therefore, it has been thought that the combination of EMS and voluntary contraction, STEMS, would optimize exercise effects more than EMS or conventional exercise alone.

In this study, we found that STEMS was superior to ST in terms of changes in core muscle thickness during voluntary contraction. Effective muscle hypertrophy was achieved in both the STEMS and ST groups based on muscle thickness at rest and during contraction. However, the contracted muscle thickness was more profound in the STEMS group, as measured by US. Contracted muscle thickness reveals a person’s ability to modulate each muscle during voluntary contraction.^[37]^ A previous review article and research stated that the improvements in muscle strength induced by STEMS are more pronounced than those induced by voluntary contraction or EMS practiced alone. Dervisevic et al also observed that isokinetic training combined with EMS induced a significant increase in muscle strength and larger adaptations compared to isokinetic training or EMS alone.^[38]^ In addition, the cross-sectional area of the target muscle was more profoundly increased in STEMS than in voluntary contraction, which is concordant with our results.^[26,39]^ It is known that EMS training increases the neural drive from the supraspinal center, resulting in greater motor unit recruitment and maximum positive neuromodulations.^[40]^ In other words, the results of this study are expected to stem from the fact that the effect of ST was maximized, as EMS has an additional positive effect on neuromodulation and adaptation of stimulated muscles. Only the contracted muscle thickness of the TrA did not differ between the 2 groups, probably because the TrA was the deepest abdominal muscle that would not be sufficiently stimulated by EMS.

In this study, we further examined the effects of EMS on physical performance including isometric muscle power, complex whole-body dynamic activity, and core stability. The 2 training methods, STEMS and ST, improved the overall physical performance after training, but no significant difference was observed between the 2 groups. Improvements in functional movement are achieved not only by strength gains but also by the amelioration of various factors such as proprioception, peak torque, range of motion, and movement patterns.^[41]^ In addition, athletic performance is affected by co-activation and coordination between agonist and antagonist muscles.^[42]^ However, EMS only stimulates the muscles beneath the attached electrodes. Any physical performance requires activation of several synergic and stabilizer muscles that are not stimulated by EMS. Even though the EMS used in the study co-stimulated the abdominal, gluteal, and hip adductor muscles, it would still be insufficient to stimulate all the muscles participating in the physical performance we examined. Therefore, EMS does not seem to facilitate the coordination of complex movements and physical performance unless it is not combined with specific dynamic movement training.^[26]^

BIA is a practical method for assessing body composition, which allows the evaluation of core body components: fat mass, skeletal muscle mass, and water. The assessment of body composition using BIA has reached an outstanding position in studies in the fields of overall health care, nutrition, and athletic sports.^[43]^ Therefore, we considered that the use of BIA would provide useful information for evaluating the effects of EMS on the body composition. As a result, skeletal muscle mass and Inbody score tended to improve in both groups after 8 weeks of intervention. Body fat mass slightly decreased in the S + E group, while it remained almost the same in the S group. However, none of the changes were significant in the within- and between-group analyses. It seems that the effects of STEMS, which focuses on the core muscles, are diluted, since BIA evaluates overall body composition, and we did not train other body parts, such as the upper extremity, during the intervention. If locoregional body composition analysis were performed by other method such as Dual-energy x-ray absorptiometry, different results could have been obtained.

The results of this study support the clinical effect of EMS on muscle activation. However, this study had several limitations. First, we only investigated the short-term effects of EMS during 8 weeks of intervention. We did not evaluate the long-lasting effects of ST and EMS on core muscle activation and physical performance. In addition, we only enrolled young healthy subjects, and therefore could not mention its effects on other populations, such as older patients with sarcopenia or other injuries. Lastly, this study only evaluated the physical performance under the isometric tests. Therefore, in-depth research regarding more dynamic values such as endurance, angular velocity, and complex coordination abilities is needed. Further studies with various populations and investigation of optimal EMS parameters, such as wave frequency and intensity, are needed to maximize the utility of EMS.

## 5. Conclusion

EMS can be considered a complementary exercise tool that induces different physiological responses from those of voluntary contraction. To our knowledge, this is the first study to comprehensively analyze the additional effects of EMS on strengthening exercises in terms of muscle contraction ability, hypertrophy, functional movements, and body composition changes. Based on this study, we conclude that EMS is safe and that superimposed EMS training on exercise programs would have additional positive effects on efficient muscle contraction. Further studies are needed to elucidate the optimal EMS parameters which can be incorporated into conventional exercise to improve muscle properties and physiological performance.

## Author contributions

**Conceptualization:** Hyun-Joon Yoo, Sejun Oh, Yongha Seo, Byung Gon Kim.

**Data curation:** Hyun-Joon Yoo, Sangsoo Park, Sejun Oh, Yongha Seo, Byung Gon Kim.

**Formal analysis:** Hyun-Joon Yoo, Sangsoo Park.

**Funding acquisition:** Sang-Heon Lee.

**Methodology:** Hyun-Joon Yoo, Sejun Oh, Yongha Seo, Byung Gon Kim.

**Supervision:** Sang-Heon Lee.

**Writing – original draft:** Hyun-Joon Yoo.

**Writing – review & editing:** Sangsoo Park, Munjeong Kang.

## Supplementary Material
