# Supplementary material for: Effects of electrical muscle stimulation on core muscle activation and physical performance in non-athletic adults: A randomized controlled trial
Source: Medicine (Baltimore). 2023 Jan 27;102(4):e32765. doi: 10.1097/MD.0000000000032765 (PMC9875983; doi:10.1097/MD.0000000000032765)
Supplement: Supplementary file 1 [file medi-102-e32765-s001.pdf]

Table, Supplemental digital content. The detailed physical training program.

Week	Exercise Program	Protocol	Borg CR-10	EMS
1-1	Breathing (draw-in maneuver)	(3” in & 3” out) x 20reps.	2	30 Hz
	Double legs inner sneezing	3” x 10reps x 2sets		
	Dead bug 1 (legs only)	10reps x 2sets		
	SLR out	10(5”)reps x 2sets		
1-2	Breathing (draw-in maneuver)	(in 3” & out 3”) x 10reps		
	Double legs Inner sneezing	5” x 10reps		
	Dead bug 1 (legs only)	10(5”)reps x 2sets		
	SLR out	10(5”)reps x 2sets		
1-3	Inner sneezing & crunch	3” x 10reps x 2sets		
	Dead bug 2 (arms & legs)	10(5”)reps x 2sets		
	Bird dog 1	10(5”)reps x 2sets		
	SLR in & out	10(5”)reps x 2sets		
2-1	Inner sneezing & crunch	3” x 10reps x 2sets	3	70 Hz
	Dead bug 1&2	10(5”)reps x 2sets		
	Bird dog 1	10(5”)reps x 2sets		
	SLR in & out	20reps x 2sets		
2-2	Inner sneezing & crunch	3” x 10reps x 2sets		
	Dead bug 1&2	10(5”)reps x 2sets		
	Bird dog 1&2	10(5”)reps x 2sets		
	SLR in & out	20reps x 2sets		
2-3	Inner sneezing & crunch	3” x 15reps x 2sets		
	Dead bug 1&2	10(5”) x 2sets		
	Bird dog 1&2	10(5”) x 2sets		
	SLR in & out	20reps x 2sets		
3	Dead bug 1	10(5”)reps x 2sets	4	
	Reverse crunch	15(5”)reps x 2sets		
	Bird dog 2	10(5”)reps x 2sets		
	Plank (front & half side)	20” x 2sets		
	SLR in & out	15reps x 2sets		
4	Bridge	30reps x 2sets	5	
	Reverse crunch	30reps x 2sets		
	Plank (front & half side)	40”x2sets & 30”x2sets		
	SLR in	20reps x 2sets		
5	Bridge	30reps x 2sets	6	
	Reverse Crunch	30reps x 2sets		
	Half side plank up down	20reps x 2sets		
	Supine SLR	20reps x 2sets		
	Front Plank	1’x2sets		
6	Mini squat side walk w/mini band	20reps x 2sets	7	
	SL bridge side move w/mini band	10reps x 2sets		
	Side plank up down w/mini band	15reps x 2sets		
	Full crunch	20reps x 2sets		
	Supine DL split	30” x 2sets		

	Front Plank	1’ x 2sets		
7-8	Mini squat side walk w/mini band	20reps x 3sets	8~9	
	Superman	20reps x 3sets		
	Side plank Standard	1’ x 3sets		
	Bicycle crunch	1’ x 3sets		

SLR = straight leg raising, SL = single leg, DL = double leg, w/ = with, ’’ = second, ’ = minute, reps = repititions
